# Association of Atherogenic Index of Plasma With Prediabetics—A Cross‐Sectional Study

**DOI:** 10.1002/edm2.70175

**Published:** 2026-02-19

**Authors:** Ali Gohar, Muhammad Husnain Ahmad, Azhar Nazir, Humza Tariq, Abdul Rehman Shahid Khan, Hamza Zaheer, Nimra Mahmood, Muhammad Zubair, Samra Zulfiqar, Masab Ali, Hanzala Zahid, Iqra Nasir

**Affiliations:** ^1^ Department of Medicine Lahore General Hospital Lahore Pakistan; ^2^ St.Tentishev Asian Medical Institute Kant Kyrgyzstan; ^3^ Department of Medicine Children Hospital Lahore Pakistan; ^4^ Lahore General Hospital Lahore Pakistan; ^5^ Ameer‐ud‐Din Medical College Lahore Pakistan; ^6^ Department of Medicine Gulab Devi Teaching Hospital Lahore Pakistan; ^7^ Department of Internal Medicine Howard University Washington DC USA; ^8^ Department of Medicine Punjab Medical College Faisalabad Pakistan

**Keywords:** atherogenic index of plasma, cardiovascular diseases, diabetes, prediabetes

## Abstract

**Background:**

Prediabetes, defined as elevated blood glucose levels below the diabetic threshold, is a worldwide concern. In Pakistan, it is highly prevalent. Preventing the progression of diabetes requires early identification of high‐risk people. The Atherogenic Index of Plasma (AIP) has been proposed as an indicator of insulin resistance and cardiovascular risk. Our study content is to explore the relationship between AIP and metabolic indicators in patients with prediabetes.

**Methodology:**

A cross‐sectional study was conducted at a tertiary care hospital after IRB approval. A total of 334 prediabetic individuals aged 18–60 years, with no history of diabetes or use of lipid/glucose‐altering medications, were included. Anthropometric, biochemical, and demographic information was gathered, including fasting glucose, HbA1c (Glycated Haemoglobin), and lipid profiles. Different AIP quartiles were assigned to the participants. ANOVA, Chi‐square, and multivariate regression with confounder adjustment were used in the statistical study.

**Results:**

Significant differences were observed across AIP quartiles. Higher AIP was associated with male gender, older age, higher BMI, lower HDL (High Density Lipoprotein), and elevated triglycerides. Fasting glucose and insulin increased with AIP, while HbA1c showed no significant variation. In multivariate analysis, HDL and triglycerides remained the strongest independent predictors of AIP.

**Conclusion:**

AIP is significantly linked with worsening metabolic indicators in prediabetics, highlighting its potential as a simple, cost‐effective screening tool. Adding AIP into routine evaluations could help early identification and prevention of diabetes and associated cardiovascular disease.

## Graphical abstract

In 334 prediabetic adults aged 18–60 years, the Atherogenic Index of Plasma (AIP) showed significant associations with worsening metabolic indicators. Higher AIP quartiles correlated with increased BMI and triglycerides and decreased HDL. AIP may serve as a simple, cost‐effective screening tool for early identification of high‐risk prediabetic individuals.

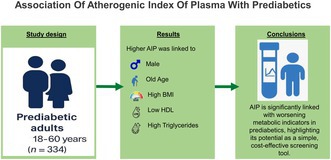

## 1 Introduction

Prediabetes is a health condition in which blood glucose level has increased over the normoglycemic levels but has not reached the glycemic range of diabetes [1]. According to WHO, the blood glucose elevation in prediabetes can be seen as an impaired fasting glucose (IFG) ranging from 110 to 125 mg/dL (6.1–6.9 mmol/L) as well as impaired glucose tolerance (IGT) with a 2‐h glucose level of 140–199 mg/dL (7.8–11.0 mmol/L) during an oral glucose tolerance test. During the prediabetes stage, the precursor of diabetes mellitus, abnormalities in glucose metabolism begin to occur, usually accompanied by insulin resistance and dyslipidemias [2, 3]. This process is a first step in a metabolic cascade which has potentially dangerous consequences. If not adequately addressed, prediabetics have a higher risk of developing diabetes than those with normoglycemia. Globally, a huge number of individuals have blood glucose levels falling in the range of prediabetes [4, 5]. The International Diabetes Federation reported that prevalence rates of prediabetes were 7.7% worldwide in 2017, which affected around 374 million people. This percentage is expected to reach 8.0% (454 million people) by 2030 and 8.6% (548 million people) by 2045 [6, 7]. A survey conducted by the Diabetes Prevalence Survey of Pakistan (DPS‐PAK) reported in 2018 the prevalence of prediabetes as 10.91% [8]. As a result of the significant strain this condition places on both patients and healthcare systems globally, there is a need for an affordable, accessible, and dependable tool to regularly detect high‐risk cases. Several studies have investigated the predictive ability of the atherogenic index of plasma (AIP), calculated as log^10^ (TG/HDL‐C), which was introduced by Dobiásová et al. in 2000 [9]. The AIP, calculated using the ratio of triglycerides to HDL cholesterol, has emerged as a valuable marker in identifying early metabolic disturbances. Elevated AIP levels have been significantly associated with insulin resistance, a key factor in the development of prediabetes. Studies have shown that individuals with higher AIP values are more likely to exhibit impaired glucose regulation and an increased risk of progressing to type 2 diabetes. Moreover, apart from enhanced risk of progression from prediabetic state to diabetes, higher AIP levels are also positively related to increased cardiovascular and metabolic syndrome development risk [10, 11]. Therefore, AIP serves as a potential early indicator for detecting prediabetes and assessing cardiovascular and metabolic risk [10, 11].

### 1.1 Rationale

Prediabetes is increasingly common in Pakistan, highlighting the need for early risk identification. The AIP has been suggested as a useful marker for metabolic disturbances, but research linking AIP to prediabetes is still limited and shows variations across different populations. To address this gap, our study explores the relationship between AIP and metabolic indicators in patients with prediabetes.

## 2 Materials and Methods

This cross‐sectional study was conducted at the Department of Internal Medicine, in a tertiary care hospital in Lahore, Pakistan, over a duration of 3 months, from February 1, 2025, to April 30, 2025. Ethical approval for the study was obtained prior to data collection from the Institutional Review Board (Reference Number: 2025/ERC/04). All ethical guidelines and protocols were strictly followed throughout the study.

The minimum required sample size was calculated to be 334, using a 95% confidence interval, with an anticipated population proportion of 0.10, and employing hypothesis testing for a population proportion. However, considering the potential for participant non‐response inherent to non‐probability convenience sampling, a total of 376 patients were initially approached for enrollment. After data collection, the final response rate was calculated to be 90%.

Patients included in the study were those visiting the internal medicine outpatient and inpatient departments during the study period and who met the inclusion criteria. Informed consent was obtained from all participants prior to inclusion in the study. Participants were thoroughly briefed on the purpose, procedures, voluntary nature, and confidentiality of the research. They were assured that refusal to participate would not affect their medical care and that they could withdraw at any point without providing a reason. Written informed consent was obtained in cases where participants were literate, while verbal consent—witnessed and documented—was used for participants who were unable to read or write.

### 2.1 Inclusion Criteria

- Adults aged 18–60 years.
- Patients diagnosed with pre‐diabetes (IFG ranging from 110 to 125 mg/dL (6.1–6.9 mmol/L) as well as IGT with a 2‐h glucose level of 140–199 mg/dL (7.8–11.0 mmol/L) during an oral glucose tolerance test (2, 3))
- No known history of diabetes mellitus.
- No current use of medications that affect glucose or lipid metabolism.

### 2.2 Exclusion Criteria

- Patients with known secondary causes of dyslipidemia (Chronic diseases).
- Patients on lipid‐lowering medications.

Data was collected using a structured questionnaire via Google form. Participants completed a questionnaire covering demographics, medical history, and lifestyle factors. Data were collected through a structured questionnaire administered by four trained researchers. These researchers were trained under the supervision of senior internal medicine faculty, who provided instruction on screening criteria for prediabetes, ethical patient approach techniques, and proper data recording practices. The training also included practical demonstrations and mock interviews to ensure standardised and accurate data collection, particularly from older patients and those admitted in inpatient settings who may have had communication or mobility limitations. The questionnaire was developed in English, translated into Urdu, and pilot‐tested to ensure clarity and relevance.

### 2.3 Statistical Analysis

Data were analysed using Statistical Package for the Social Sciences (SPSS) version 26.0. Descriptive statistics were applied to calculate frequencies and percentages for categorical variables, and means with standard deviations for continuous variables. Chi‐square tests and ANOVA, were employed where necessary to determine associations, with a significance threshold (*α*) set at *p* < 0.05. ANOVA was used to compare mean values of continuous variables (e.g., AIP, glucose levels) across multiple demographic or clinical subgroups. The Chi‐square test was applied to assess associations between categorical variables (e.g., gender, comorbidities, AIP risk categories) and prediabetic status.

## 3 Results

The study investigated the association of AIP with various demographic, anthropometric, and biochemical parameters among individuals with prediabetes. Participants were divided into four quartiles based on their AIP values (< 0.14, 0.14–0.33, 0.33–0.56, > 0.56), and significant variations were observed across these groups.

In terms of gender distribution, there was a statistically significant difference between quartiles (*p* = 0.000), with a higher proportion of males in higher AIP categories, suggesting a gender‐related risk for atherogenic disease. Age also increased significantly with rising AIP levels (*p* = 0.006), indicating that older individuals tend to have higher atherogenic indices.

Anthropometric measures like height, weight, and BMI (Body Mass Index) showed clear differences between the different AIP groups, with the group having the highest AIP also having higher weight and BMI. This supports the well‐known link between obesity and a greater risk of heart disease.

The biochemical results revealed some clear patterns. HDL cholesterol dropped sharply as AIP levels increased—from an average of 131.5 mg/dL in the lowest group to just 41.7 mg/dL in the highest (*p* = 0.000). At the same time, LDL (Low Density LipoProtien), total cholesterol, and triglyceride levels rose steadily across the AIP quartiles (all *p* = 0.000). These trends are expected, given that AIP is based on the ratio of triglycerides to HDL, making it a direct reflection of worsening lipid profiles.

Interestingly, fasting insulin and glucose levels varied significantly across the AIP groups (*p* = 0.000), with higher fasting glucose seen in the groups with higher AIP. This pattern fits with the metabolic imbalances often seen in people with prediabetes. On the other hand, HbA1c levels did not show much difference between the groups (*p* = 0.300), which suggests that while AIP is closely linked to lipid and short‐term glucose changes, its connection to long‐term blood sugar control might be weaker or possibly influenced by other factors in prediabetic individuals (Table 1).

**TABLE 1:** Baseline characteristics of the study participants.

Variable	Total		AIP quartiles				
			< 0.14	0.14–0.33	0.33–0.56	> 0.56	*p* value
Gender	Male	143 (42.8%)	22	14	48	58	0.000
	Female	191 (58.2%)	49	29	64	49	
Age (years)	Mean	40.263	37.861	36.558	41.893	41.664	0.006
	S.D.	1.0428	10.4414	11.6666	10.4592	11.2828	
Height (cm)	Mean	163.901	160.375	163.349	162.643	167.813	0.000
	S.D.	105.229	12.3236	11.9720	8.9753	8.9053	
Weight (kg)	Mean	73.461	68.069	70.349	75.402	76.308	0.000
	S.D.	11.9444	11.1461	12.6301	11.4612	11.3307	
HbA1c (%)	Mean	6.073	6.009	6.026	6.067	6.081	0.300
	S.D.	0.2056	0.2120	0.2060	0.1793	0.2253	
HDL (mg/dL)	Mean	72.940	131.500	100.302	54.643	41.692	0.000
	S.D.	37.4180	18.8978	17.8132	5.9878	4.8495	
LDL (mg/dL)	Mean	101.479	48.722	43.767	132.214	128.000	0.000
	S.D.	43.1609	7.1527	5.3977	23.9949	15.9285	
Cholesterol (mg/dL)	Mean	204.916	198.389	192.674	207.589	211.430	0.000
	S.D.	21.5679	23.2957	27.9606	17.1388	18.4494	
Triglycerides (mg/dL)	Mean	163.389	133.542	158.837	169.107	179.318	0.000
	S.D.	25.5942	21.9448	28.1915	16.4899	15.2146	
Insulin (μIU/mL)	Mean	16.075	17.353	17.100	16.450	14.411	0.000
	S.D.	5.0598	4.4430	4.7116	5.0172	5.2508	
Fasting Glucose (mg/dL)	Mean	109.263	105.750	103.651	111.486	111.243	0.000
	S.D.	13.3483	18.6485	21.4675	7.4500	7.3649	
BMI (kg/m^2^)	Mean	27.632	26.8875	26.8000	28.82593	27.2198	0.041
	S.D.	5.4844	5.75054	6.40167	5.71530	4.42322	

The multivariate regression analysis provided deeper insight into these relationships. Even after accounting for age, gender, and BMI, factors like age (*p* = 0.020), gender (*p* = 0.024), and BMI (*p* = 0.011) still showed a significant link with AIP levels. Most notably, HbA1c (*p* < 0.001) stood out as a strong predictor. These results suggest that each of these factors independently plays a role in identifying cardiovascular risk among people with prediabetes (Tables 2, 3, 4).

**TABLE 2:** Model fitting information (unadjusted).

Model	Model fitting criteria	Likelihood ratio tests		
	−2 log likelihood	Chi‐square	df	Sig.
Intercept only	151.189			
Final	80.487	70.702	24	0.000

**TABLE 3:** Model fitting information (adjusted for age, gender, BMI).

Model	Model fitting criteria	Likelihood ratio tests		
	−2 log likelihood	Chi‐square	df	Sig.
Intercept only	882.530			
Final	780.854	101.676	33	0.000

**TABLE 4:** Likelihood ratio tests.

Effect	Model fitting criteria	Likelihood ratio tests		
	−2 log likelihood of reduced model	Chi‐square	Df	Sig.
Intercept	780.854^a^	0.000	0	0
Age	790.738	9.885	3	0.020
Gender	790.326	9.473	3	0.024
BMI	792.020	11.166	3	0.011
HbA1c	849.852	68.998	24	0.000

^a^ The intercept represents the baseline model. No likelihood ratio test is performed because there is no reduced model for comparison.

After adjusting for various factors like HDL, LDL, cholesterol, triglycerides, insulin, and fasting glucose, only HDL and triglycerides remained statistically significant (both with *p* = 0.000). This finding confirms that these two lipid components are the main contributors to changes in AIP and are strong, reliable indicators for assessing a person's risk of developing atherosclerosis.

The model diagnostics showed a good overall fit, with statistically significant results from the likelihood ratio tests (*p* = 0.000) in both the unadjusted and adjusted models. However, the fully adjusted model faced some technical issues, such as mathematical errors in the calculations (singularities in the Hessian matrix). These problems may have been caused by overlapping variables (multicollinearity) or small group sizes within the data. This suggests that the model may need to be simplified or refined in future studies to improve its stability and reliability (Tables 5 and 6).

**TABLE 5:** Model fitting information (adjusted for age, gender, BMI, HDL, LDL, cholesterol, triglycerides, insulin and fasting glucose levels).

Model	Model fitting criteria	Likelihood ratio tests		
	−2 log likelihood	Chi‐square	df	Sig.
Intercept Only	882.530			
Final	0.005	882.525	51	0.000

**TABLE 6:** Likelihood ratio tests.

Effect	Model fitting criteria	Likelihood ratio tests		
	−2 log likelihood of reduced model	Chi‐square	Df	Sig.
Intercept	0.005^a^	0.000	0	0
Age	0.005^b^	0.000	3	1.000
Gender	0.005^b^	0.000	3	1.000
BMI	0.006^b^	0.001	3	1.000
HDL (mg/dL)	328.443^b^	328.438	3	0.000
LDL (mg/dL)	0.015^b^	0.010	3	1.000
Cholesterol (mg/dL)	0.006^b^	0.001	3	1.000
Triglycerides (mg/dL)	147.621^b^	147.616	3	0.000
Serum Insulin (μIU/mL)	0.005^b^	0.000	3	1.000
Fasting Glucose (mg/dL)	0.006^b^	0.001	3	1.000
HbA1c	0.000^c^	0	24	0

^a^ The intercept is a baseline reference and is not tested for significance using a likelihood ratio test.

^b^ This row represents a standard likelihood ratio test comparing reduced and full models.

^c^ This reduced model is equivalent to the full model or the parameter estimates are redundant; therefore, the likelihood ratio test cannot be computed.

**FIGURE 1:**
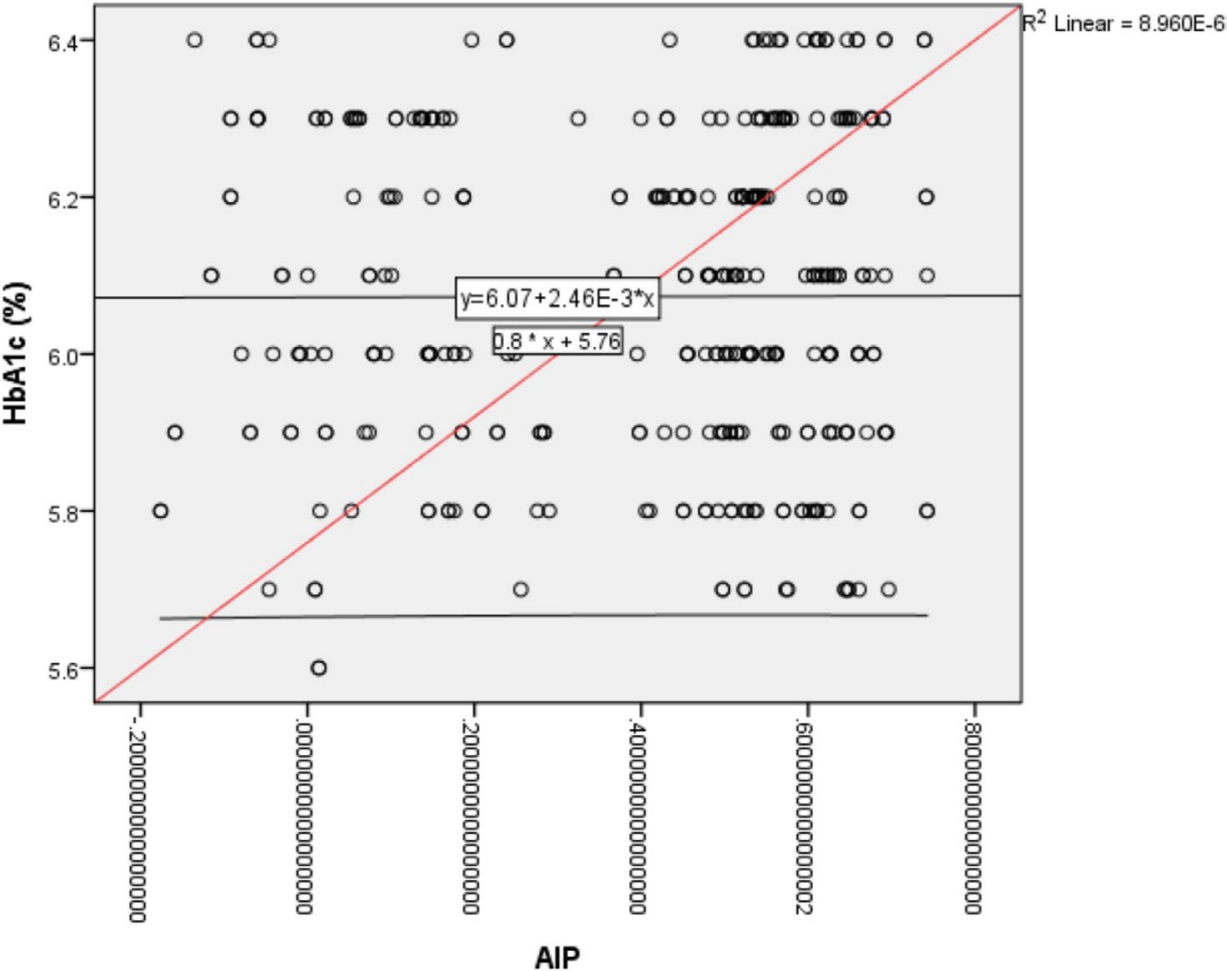
Non‐Linear Relationship between HbA1c (Glycated Haemoglobin) and Atherogenic Index of Plasma (AIP).

Overall, the findings clearly show that higher AIP levels are closely linked to a poorer cardiometabolic profile, particularly marked by abnormal lipid levels and signs of insulin resistance. The strong relationship between AIP, HDL cholesterol, and triglycerides highlights AIP's value as a simple, affordable, and reliable marker for spotting early cardiovascular risk in people with prediabetes. These results suggest that including AIP in routine health screenings—especially for those at higher risk—could help identify problems early and allow for timely steps to prevent the development of full‐blown diabetes and heart disease (Figure 1).

## 4 Discussion

This cross‐sectional study assessed the relationship between AIP and prediabetes, aiming to identify a possible early indicator for metabolic risk in a population with high prevalence. Although this was a single‐center, hospital‐based study, the study population represents a diverse group of patients commonly encountered in tertiary care settings in Pakistan. Individuals from varied socioeconomic and urban–rural backgrounds increase the external relevance of the findings. However, caution should be exercised when generalising the results to the entire Pakistani population. Multicenter and community‐based studies across different regions are needed to further validate the applicability of AIP as a screening marker at the national level. Future studies should include diverse populations and longitudinal designs to better understand the AIP‐insulin resistance relationship in prediabetic patients.

Given the rising frequency of prediabetes worldwide, particularly in Pakistan, where 10.91% of the population was reported to have the disease in 2018 [8], our findings have important clinical implications. Identifying an accessible and cost‐effective risk marker, such as AIP, could provide a practical way to detect individuals at risk before they develop diabetes [9]. The AIP, which is based on the triglyceride‐to‐HDL ratio, is a valuable indicator for early signs of metabolic issues. Elevated AIP may be an early indicator of prediabetes and associated cardiovascular issues because it is linked to insulin resistance and an increased risk of blood sugar imbalance [10, 11].

Prediabetes is increasingly recognised as a metabolically adverse state associated with early insulin resistance, dyslipidemia, and increased cardiovascular risk, even before the onset of overt diabetes [9, 10, 11, 12]. Because of its dependability and independence from glucose‐based tests, AIP is a useful substitute for the screening of cardiovascular problems related to prediabetes. Although HbA1c is frequently utilised for diagnosing prediabetes, its drawbacks and expense render alternative markers such as AIP attractive, particularly in settings with limited resources. Since maintaining lifestyle modifications can be challenging, utilising straightforward, early indicators like AIP could assist in identifying individuals requiring prompt interventions [2].

In our study, participants were divided into different AIP quartiles to evaluate trends in their metabolic and lipid profiles. The previous studies did not categorise AIP into quartiles for a stepwise analysis; most results were either qualitative or categorised based on the presence or absence of MetS traits. The majority of the studies focus on blood glucose levels or HbA1c, but few look at lipid‐based markers like AIP. Early detection of cardiovascular risk in people with prediabetes is understudied. There is also a shortage of data from South Asian populations, who are at higher risk for prediabetes and heart disease [3].

Other researchers identified particular thresholds for AIP (for instance, < 0.04 to reverse prediabetes), but our novel study highlights and points towards the necessity for more exploration into AIP cutoffs, especially for South Asian groups.

Unlike our study, previous literature concentrated more on progression and regression but did not thoroughly explain the baseline characteristics across different AIP levels. Our study explained and particularly revealed the trends related to BMI, TG, HDL, glucose, and more [10].

Research also indicates that the hormone resistin rises in prediabetes, highlighting early signs of insulin resistance. The distinctions between Impaired Fasting Glucose (IFG) and Impaired Glucose Tolerance (IGT) imply that prediabetes is not a singular condition, and AIP levels may differ among these subgroups [1].

Another study found that age‐based subgroup analysis makes AIP a more reliable predictor in younger populations. Earlier research suggests that AIP might be a more accurate predictor in younger people with prediabetes, which we did not examine in our study but could look into in future subgroup analyses [3].

Male gender, advanced age, elevated BMI, and bad lipid profiles were significantly linked to elevated AIP levels in prediabetic people. Triglycerides raised and HDL levels decreased as AIP increased; fasting glucose and insulin levels also changed. The significance of AIP as a simple marker for early cardiometabolic risk was highlighted by multivariate analysis, which confirmed HDL and triglycerides as the main drivers [1].

People with prediabetes often have higher BMI, lower HDL, and higher triglycerides, which are early markers of cardiovascular risk. The concept of using AIP as an indication to detect metabolic problems before the development of full‐blown diabetes is supported by these variations [1]. Summary of key studies on AIP and its association with various conditions is shown in (Table 7).

**TABLE 7:** Summary of key studies on AIP and its association with various conditions.

Study (first author, year)	Design/population	Condition(s) studied	Key findings on AIP	Conclusion
Lioy et al. [9]	Narrative review	Cardiometabolic risk factors	Summarised strong evidence linking elevated AIP with dyslipidemia, metabolic syndrome, insulin resistance, hypertension, and atherosclerosis	AIP is a reliable integrative marker for cardiometabolic risk assessment
Wang et al. [13]	Longitudinal CHARLS cohort (China), adults with cardiovascular‐kidney‐metabolic syndrome (stages 0–3)	Stroke risk	Higher baseline and modified AIP (including AIP‐BMI, AIP‐WC/WHtR) predicted increased incident stroke over time	AIP and its variations are significant predictors of stroke in individuals with cardiometabolic multimorbidity
Zou et al. [12]	Prospective CHARLS cohort, middle‐aged & elderly Chinese	Development of prediabetes	Cumulative higher AIP exposure associated with increased risk of developing prediabetes	Longitudinal monitoring of AIP can help identify individuals at risk for prediabetes
Du et al. 2024 [14]	Cross‐sectional NHANES (USA), ~4841 adults	Gallstones	AIP partly mediated the link between higher dietary magnesium intake and lower gallstone prevalence (indirect effect size 0.003, 95% CI 0.001–0.005)	AIP is a significant mediator between nutrition and gallstone risk
Huang et al. 2024 [15]	CHARLS cohort, adults ≥ 45 years (*n*≈3791)	Cardiometabolic diseases (CMD): diabetes, hypertension, Cardiovascular disease	Greater increases in AIP were linked to higher CMD incidence; gender‐specific effects with stronger associations in men for some outcomes	Regular AIP monitoring may help stratify CMD risk in older adults, with sex‐specific considerations
Bikov et al. 2021 [16]	Observational cross‐sectional, 461 OSA patients vs. 99 controls (Europe)	Obstructive Sleep Apnea (OSA) severity & cardiovascular risk	AIP significantly higher in OSA patients and correlated with disease severity indicators	AIP may serve as a lipid‐related biomarker for cardiovascular risk in OSA

The issues encountered in model fitting indicate that there is a need for refined statistical modelling in future research. Our study is observational; since this is a cross‐sectional study, it only shows a snapshot in time, so we cannot tell whether one thing can cause the other, and the findings may not apply to all populations. Also, given the cross‐sectional design of this study, temporal relationships and causality between AIP and metabolic abnormalities cannot be established, which should be considered a mild limitation.

Our study shows that AIP is a straightforward and affordable way to detect metabolic risk early in people with prediabetes. By looking at AIP quartiles, we observed clear patterns in BMI, TG, HDL, and glucose levels. The results suggest that AIP is closely linked to heart and metabolic risk factors, especially in men, older adults, and those with unhealthy cholesterol profiles. These findings support the idea that AIP could be a useful screening tool, especially in areas with limited resource settings.

## 5 Conclusion

This study demonstrates a significant association between the AIP in prediabetic individuals, particularly elevated triglycerides, reduced HDL, higher BMI, and increased fasting glucose and insulin levels. These findings support AIP as a simple, cost‐effective marker that can be integrated into routine screenings to identify individuals at high risk for progressing to diabetes and cardiovascular disease. Notably, AIP showed stronger correlations than HbA1c with short‐term metabolic changes, suggesting its added value in early detection. Future longitudinal studies, especially in South Asian populations, are warranted to establish AIP cut‐off thresholds, validate its predictive capacity, and explore its utility across different prediabetes phenotypes.

## Author Contributions

**Ali Gohar:** conceptualization, validation, visualisation. **Muhammad Husnain Ahmad:** writing – original draft, writing – review and editing, visualisation, validation. **Azhar Nazir:** investigation, writing – original draft, writing – review and editing. **Humza Tariq:** writing – original draft, conceptualization, methodology. **Abdul Rehman Shahid Khan:** investigation, visualisation, validation, methodology. **Hamza Zaheer:** writing – review and editing, writing – original draft, validation. **Nimra Mahmood:** writing – review and editing, methodology, conceptualization. **Muhammad Zubair:** investigation, visualisation, writing – original draft. **Samra Zulfiqar:** validation, visualisation, conceptualization. **Masab Ali:** validation, visualisation. **Hanzala Zahid:** writing – original draft, writing – review and editing, validation. **Iqra Nasir:** validation, visualisation, investigation.

## Conflicts of Interest

The authors declare no conflicts of interest.

## Data Availability

Data sharing is not applicable to this article as no new data were created or analyzed in this study.
